# Uncovering the mechanism of Qidan Dihuang Granule in the treatment of diabetic kidney disease combined network pharmacology, UHPLC-MS/MS with experimental validation

**DOI:** 10.1016/j.heliyon.2023.e21714

**Published:** 2023-10-28

**Authors:** Lei Xiang, Xiangsheng Cai, Xiaoshan Zhao, Yuanling Liu, Ya Xiao, Pingping Jiang, Lianghong Yin, Dan Song, Xuefeng Jiang

**Affiliations:** a Department of Nephrology Internal Medicine, The First Affiliated Hospital of Jinan University, 510630, Guangzhou, China; b Clinical Laboratory, Guangzhou Cadre Health Management Center, Guangzhou No.11 People's Hospital, Guangzhou, 510530, China; c School of Traditional Chinese Medicine, Southern Medical University, 510515, Guangzhou, China; d Administrative Department, Guangdong Women and Children Hospital, 510010, Guangzhou, China; e School of Traditional Chinese Medicine, Jinan University, 510632, Guangzhou, China; f Department of Traditional Chinese Medicine, The First Affiliated Hospital of Guangdong Pharmaceutical University, 510062, Guangzhou, China; g Department of Nephrology Internal Medicine, University of Chinese Academy of Science-Shenzhen Hospital, 518107, Shenzhen, China

**Keywords:** Qidan Dihuang Granule, Diabetic kidney disease, Network pharmacology, Experimental verification, Traditional Chinese medicine

## Abstract

**Background and aim:**

Diabetic Kidney Disease (DKD) is a common microvascular complication of diabetes mellitus. Multi-center, randomized controlled trials have shown that Qidan Dihuang Granule (QDDHG) reduces the levels of albuminuria of DKD. However, the specific mechanisms of QDDHG on DKD are not clarified. Thus, this study utilized network pharmacology, UHPLC-MS/MS (Ultra-High Performance Liquid Chromatography - Mass Spectrometry) and animal experiments to reveal the mechanisms of QDDHG on DKD.

**Experimental procedure:**

Screening and retrieving active ingredients and corresponding targets of QDDHG on DKD through the TCMSP, ETCM, Disgenet, GeneCards, Omim and DrugBank databases. The PPI were performed with BioGrid, STRING, OmniPath, InWeb-IM. AutoDock Vina molecular docking module to estimate the validation from the compounds and target proteins. Free energy to estimate the binding affinity for identified compounds and target proteins. The ingredients of QDDHG were analyzed utilizing UHPLC-MS/MS. *In vivo* experiment with db/db mice were used to verify the targets and pathway predicted by network pharmacology.

**Results and conclusion:**

The results demonstrated that QDDHG has 18 active compounds and 13 target proteins of QDDHG exerted a crucial role in treatment of DKD. QDDHG affect the multiple biological processes included cellular response to lipid, response to oxidative stress, and various pathways, such as AGE-RAGE, PI3K-Akt, MAPK, TNF, EGFR, STAT3. The results of UHPLC-MS/MS showed that six ingredients predicted by network pharmacology were also verified in experiment. *In vivo* experiment verified the effects of QDDHG on protecting the renal function mainly through inhibited the expression of EGFR, STAT3 and pERK in the db/db mice.

## Highlights

• This work is the first to reveal the mechanism underlying the effect of QDDHG on DKD based on RCT results.
• QDDHG was studied using network analysis, UHPLC-MS/MS, and animal experiments.
• The mechanism of QDDHG involved inhibition of the expression of EGFR, STAT3 and pERK in DKD mice.
• This study demonstrates that TCM exerts its the effects through multiple components and pathways.

## Abbreviations

AGE: advanced glycosylation end
Alb: Urine albumin
BC: betweenness centrality
BUN: blood urea nitrogen
CC: closeness centrality
COX-2: mitochondrially encoded cytochrome *c* oxidase II
CTGF: connective tissue growth factor
DKD: diabetic kidney disease
DL: drug-likeness
DN: Diabetic Nephropathy
ELISA: Enzyme-linked immunosorbent assay
ERK1/2: Extracellular signal-regulated protein kinase 1/2
ESRD: end-stage renal disease
ETCM: The Encyclopedia of Traditional Chinese Medicine
GO: Gene Ontology
HE: hematoxylin-eosin
HIF-1: Hypoxia-inducible factor 1
HPLC: High-performance liquid chromatography
IKBa: NFKB inhibitor alpha
IL-17: Interleukin 17
IL-1β: Interleukin 1 beta
IL22: Interleukin 22
IL-6: Interleukin 6
KEGG: Kyoto Encyclopedia of Genes and Genomes
MAPK: Mitogen-activated protein kinase
Masson: Masson's trichrome
MS: Mass Spectrometry
NC: Normal Control
NF-kappa B: Nuclear factor-kappa B
OB: Oral bioavailability
PAS: Periodic acid-Schiff
PDB: Protein Data Bank
pERK1/2: Phospho-extracellular signal-regulated protein kinase 1/2
PI3K: Phosphoinositide 3-kinases
PPAR: Peroxisome proliferator-activated receptor
PPI: protein-protein interaction
QDDHG: Qidan Dihuang Granules
RAGE: advanced glycosylation end-product specific receptor
Rap1: Ras-related protein 1
ROS: reactive oxygen species
Scr: Serum creatinine
SD: Standard Deviation
SIRT1: sirtuin 1
STRING: Search Tool for the Retrieval of Interacting Genes/Proteins
TCM: traditional Chinese medicine
TCMNPAS: TCM Network Pharmacology Analysis System
TCMSP: Traditional Chinese Medicine Systems Pharmacology Database and Analysis Platform
TEM: Transmission Electron Microscope
TGF-β1: transforming growth factor beta 1
TNF: Tumor necrosis factor
UHPLC-MS/MS: Ultra-High Performance Liquid Chromatography - Mass Spectrometry
VEGF: vascular endothelial growth factor

## 1 Introduction

Diabetic Kidney Disease (DKD) is one of common microvascular complication of diabetes mellitus [1] and has become the principal cause of ESRD (End-stage renal disease) worldwide [2], with about 50 % of patients with DKD likely to develop ESRD [3]. At present, the treatment of DKD is not entirely effective in blocking kidney damage [4]. Previous studies have shown that Traditional Chinese medicine (TCM) treatment was contributed to delay the decline of renal function and decreased risk of ESRD and mortality rate among patients with chronic kidney disease and diabetes mellitus [5,6]. Under the guidance of the TCM theory [[7], [8], [9]], the Qidan Dihuang Granules (QDDHG) including Huangqi (*Astragalus mongholicus* Bunge), Danshen (*Salvia miltiorrhiza* Bunge), Dihuang (*Rehmannia glutinosa* (Gaertn.) DC.), Shanyao (*Dioscorea oppositifolia* L.), and Gancao (*Glycyrrhiza echinata* L.), were proposed to treat DKD. Multi-center, randomized controlled trials (RCT) have demonstrated that QDDHG combined with Angiotensin Receptor Blockers administration can significantly lower the levels of albuminuria, improve the TCM syndrome score, and delay the disease process [10].

However, the specific mechanisms of QDDHG on DKD are not clarified. Unlike synthesized compounds or single herbal extracts, QDDHG has multiple components, and maybe performs systemic function via multiple pathways in treatment of DKD. Traditional studies’ method concentrating on specific mechanisms may have shortcoming for the assessment of mechanisms owing to QDDHG with multiple components and targets. As a systemic research method, network pharmacology could identify the potential mechanisms and target proteins of TCM for subsequent verification *in vivo* and *in vitro* studies. Therefore, this study utilized network pharmacology methods to retrieve active compounds and corresponding genes of QDDHG, and targets of DKD, and reveal the potential therapeutic mechanisms. Furthermore, molecular docking was applied to verify putative targets. Finally, identification of the chemical components of QDDHG was performed using UHPLC-MS/MS and animal experiments were used to verify the targets and pathway predicted by network pharmacology methods.

## 2 Methods

### 2.1 Exploration of mechanism based on network pharmacology

#### 2.1.1 Screening of active ingredients and corresponding potential targets of QDDHG

The TCMSP (https://tcmsp-e.com/) and ETCM (http://www.tcmip.cn/ETCM/) database were accessed, and the active ingredients of QDDHG were retrieved. Oral bioavailability (OB) ≥ 30 % and Drug-Likeness (DL) ≥ 0.18 or Drug-likeness Grading of “good” were set as the thresholds for identifying active ingredients [11,12]. The active compounds were identified in the UniProt (https://www.uniprot.org/), with the screening conditions “homo sapiens” and “reviewed”. For ingredients not included in the TCMSP and ETCM, the Swiss Target Prediction database (http://swisstargetprediction.ch/) was searched to identify the corresponding genes.

#### 2.1.2 Screening of DKD targets

Screening targets of DKD was carried out by searching the Disgenet (https://www.disgenet.org/), Omim (http://www.omim.org/), GeneCards (https://www.genecards.org/), and DrugBank (https://go.drugbank.com/) databases with the terms “DN” (diabetic nephropathy) or “DKD” (diabetic kidney disease).

#### 2.1.3 Putative targets of QDDHG on DKD and construction of PPI network

A Venny diagram was constructed using the common genes of QDDHG and DKD. The PPI enrichment of intersecting targets were performed with the Metascape databases (https://metascape.org/gp/). Then, Cytoscape 3.6.1 was used to construct a PPI network diagram, and topology analysis was carried out with the “Network Analyzer” plug-in network structure. “CC”, “BC”, and “Degree” were calculated to discover core targets [13].

#### 2.1.4 GO and KEGG pathway enrichment on putative targets

GO and KEGG analyses were constructed using Metascape. The Cutoff values were set as follows: *P*-value cutoff equal to 0.01, Min Overlap equal to 3, and Min Enrichment equal to 1.5. Enriched GO and KEGG pathways were visualized using an online bioinformatics platform (http://www.bioinformatics.com.cn/).

#### 2.1.5 Screening of core active compounds by construction of herb- active compounds-targets-pathways network

The experimental data, including the herb, active compounds, targets, and KEGG pathways were input to Cytoscape 3.6.1 to establish an herb-compounds-targets-pathways network. Network Analyzer was used to filter the core chemical components with BC ≥ median, CC ≥ median, and degree≥ 2 times the median degree.

### 2.2 Molecular docking verification

The built-in AutoDock Vina version 1.1.2 docking module of the TCMNPAS online analysis platform was used for molecular docking verification [14]. The small molecules were input in SMILES format, the protein receptor ID number was obtained from the PDB database (https://www.rcsb.org/), and the default Vina compound ligand preparation program was used [15]. The protein standard ligand molecule file was obtained through the “Extract standard ligand molecule from PDB” function of the utility module. The docking algorithm uses the built-in PSOVina Version 1.0 tool, and “From Ligand” was selected for the interface bag parameters.

### 2.3 Experimental verification

#### 2.3.1 Chemicals, UHPLC-MS/MS, and main reagents

The QDDHG formula used in this study was of Chinese granule herbal extracts. The grains were purchased from Tianjiang Pharmaceutical Co., Ltd. (Jiangyin, Jiangsu, China). The herbal composition is shown in Table 1. Component analysis of QDDHG was performed using an UltiMate 3000 liquid chromatography coupled with Q Exactive mass spectrometer (Thermo scientific, CA, USA) system. Primary antibodies against activating transcription EGFR, STAT3, and β-actin were purchased from Servicebio Technology Co., Ltd. (Wuhan, China). ERK1/2 and phosphorylated ERK1/2 (pERK1/2) were purchased from Cell Signaling Technology.

**Table 1:** The herbal composition and proportion of QDDHG.

Chinese name	Scientific name	Pinyin name	Plant part used	Batch number	Botanical drug dose (g)	Composition (%)	Validated compounds	Targets
Huangqi	*Astragalus mongholicus* Bunge	huáng qí	Root	20120653	75	37.5	17	204
Danshen	*Salvia miltiorrhiza* Bunge	dān shēn	Root	21010213	37.5	18.75	59	132
Dihuang	*Rehmannia glutinosa* (Gaertn.) DC.	dì huáng	Root	21010143	37.5	18.75	8	256
Shanyao	*Dioscorea oppositifolia* L.	shān yào	Root	20081863	37.5	18.75	15	68
Gancao	*Glycyrrhiza echinata* L.	gān cǎo	Root	21010043	12.5	6.25	91	261

#### 2.3.2 Experimental animals and design

The use and care of animals and experimental protocols was processed in accordance with European Community guidelines of experimental animals, and were approved by the Ethics Committee of the University of Chinese Academy of Science-Shenzhen Hospital (Ethics number: LL-KT-2021231). Male Lepr ^db^ (db/db) mice and their non-diabetic Lepr ^db/m^(db/m) male mice were purchased from the Changzhou Cavens Laboratory Animal Co. Ltd. (Changzhou, China). All mice were raised in SPF environment, after an acclimation period of 14 days, 8-week-old mice randomly assigned to four groups. The mice of the QDDHG1 group were fed a chow diet containing 1 % QDDHG (containing 10 g of QDDHG per 1000 g of the chow diet). The mice of the QDDHG2 group were fed a chow diet containing 2 % QDDHG (containing 20 g of QDDHG per 1000 g of the chow diet). The NC (Normal Control) group consisted of the db/m mice, and the DKD group consisted of the db/db mice, which were fed a drug-free chow diet. Fasting body weight (FBW) and caudal vein fasting blood glucose (FBG) were measured at regular intervals over a 12-h fasting period. After 16 weeks of treatment, the mice were sacrificed, and the blood samples and renal tissue were collected.

#### 2.3.3 Measurement of blood and urine samples

Blood was centrifuged at 5000 rotation per minute for 10 min, and serum samples isolated. Serum creatinine (Scr) and blood urea nitrogen (BUN) were tested using a Hitachi-7600 Automatic Biochemical Analyzer (Tokyo, Japan) using the recommended procedures. 24-hour urine albumin (Alb) levels were determined using a commercially available ELISA kit (Nanjing Jiancheng, Nanjing, China).

#### 2.3.4 Renal morphology and ultrastructure assessment

Renal tissue was embedded in paraffin after fixation in 4 % (w/v) paraformaldehyde. Tissue sections were stained with hematoxylin-Eosin (HE), Periodic Acid-Schiff (PAS) and Masson's trichrome (Masson). Glycogen intensity and collagen fiber intensity in the glomerular mesangial region were calculated on PAS-stained and Masson-stained tissue, respectively, using NIS-Elements Viewer (NIKON). The PAS-positive material was identified by its purple-red color, while the nucleus was stained blue. A NIS-Elements Viewer was utilized to measure the area of the PAS-positive material and the total glomerular area. The volume ratio of glomerular glycogen was calculated as the PAS-positive material area/glomerular area. For Masson trichrome staining, the blue collagen deposition was taken as positive material, and the ratio of collagen accumulation was calculated as the Masson -positive material area/glomerular area. The ultrastructure of glomeruli, renal tissue was examined under a Tecnai Transmission Electron Microscope (TEM). The cortical portion of the renal tissue was sliced into small sections of 1 mm^3^ and transferred into an EP tube containing fresh TEM fixative. The tissue was then fixed at 4 °C for preservation and sent to Wuhan Servicebio Technology Co. Ltd. (China).

#### 2.3.5 Immunofluorescence staining

Sections of kidney tissue were deparaffinized, rehydrated, and submitted to antigen retrieval. Then, the tissue sections were covered with 3 % BSA to block non-specific binding for 30 min. The slides were incubated with primary antibody (Nephrin, Servicebio, GB11343, 1:1000 dilution) overnight at 4 °C; incubation was performed in a wet box containing a small amount of water. Next, the slides were treated with Alexa Fluor 488-conjugated goat anti-rabbit IgG (Servicebio, GB25303, 1:500 dilution) as the secondary antibody. They were then incubated with DAPI solution at room temperature for 10 min, in the dark. Next, spontaneous fluorescence quenching reagent was added, and the slides were incubated for 5 min. The slides were then cover-slipped with anti-fade mounting medium. Finally, microscopy detection was performed, and images were collected with a fluorescent microscope (NIKON ECLIPSE C1, DS-U3). The nucleus was stained blue with DAPI and positive cells were labeled green.

#### 2.3.6 Western blot analysis

Renal tissue was lysed in RIPA buffer supplemented (Roche, Canada), then, the protein quantification with the Bradford assay (Sigma, USA). Subsequently, 50 ng protein solution was electrophoresed via 7.5%–10 % SDS-PAGE (Bio-Rad, USA), transferred to PVDF membrane (Pierce, USA), blocked with 5 % BSA and incubated with antibodies (anti-EGFR, anti-STAT3, anti-β-actin at 1:2000 dilution, anti- ERK1/2, phosphorylated ERK1/2 at 1:5000 dilution) overnight at 4 °C. Next, membranes were washed and incubated with goat anti-rabbit IgG (Abcam, USA) at 1:5000 dilution for 1 h. Blots were rinsed in PBS containing 0.2 % Tween 20, three times for 5 min each at room temperature, and an enhanced chemiluminescence reagent to visualize the protein bands (Millipore, Billerica, MA, USA). The images were captured with Tanon-5200 (Tanon, China) and quantified with the image analysis software Image J (MD, USA).

### 2.4 Statistical analysis

Statistical tests were performed using SPSS 19.0. Statistical analysis was carried out using ANOVA, followed by Tukey's post hoc test or Tamhane's T^2^. Significant differences were considered when the *P*-values <0.05.

## 3 Results

### 3.1 Active compounds, putative targets of QDDHG and DKD targets

**Fig. 1:**
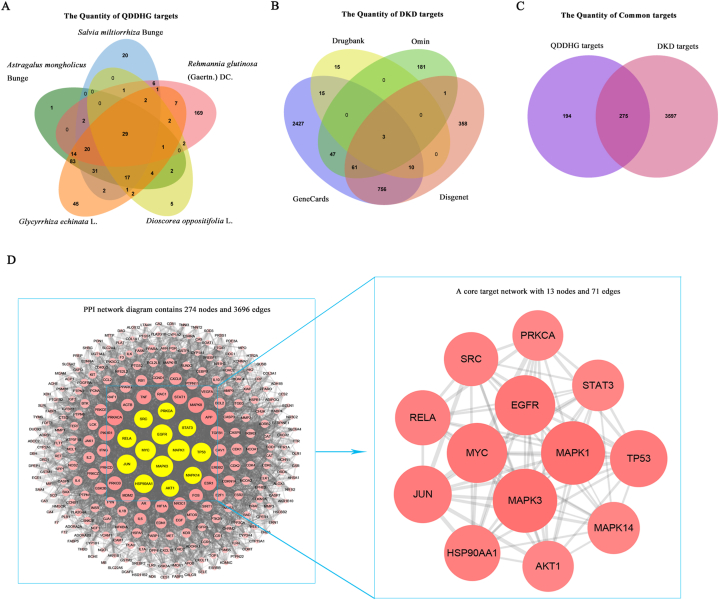
Venn plots showing the target numbers of QDDHG and DKD, and Protein–protein interaction (PPI) network. **(A)** 469 potential therapeutic targets identified from 190 active compounds of QDDHG. **(B)** 3872 genes related to DKD from 4 disease databases. **(C)** 275 genes intersecting targets between QDDHG and DKD. **(D)** PPI network.

There were 190 active compounds that meet the screening criterion, of which, Huangqi had 17 active compounds, Danshen had 59 active compounds, Dihuang had 8 active compounds, Shanyao had 15 active compounds, and Gancao had 91 active compounds. The 190 compounds corresponded to 921 genes, of which, the active compounds of Huangqi corresponded to 204 genes, Danshen corresponded to 132 genes, Dihuang corresponded to 256 genes, Shanyao corresponded to 68 genes, and Gancao corresponded to 261 genes (Table 1, Fig. 1 A, Supplementary Table S1). After removing the duplicate genes, 469 genes were retained (Supplementary Table S2).

The number of DKD genes in each database was 3319 (GeneCards), 43 (DrugBank), 293 (Omim), and 1189 (Disgenet). After removing the duplicates, 3872 genes were retained (Fig. 1 B, Supplementary Table S3).

### 3.2 Thirteen core target of QDDHG on DKD

In total, 275 putative targets were identified by intersecting the genes corresponding to the active compounds and DKD (Fig. 1C, Supplementary Table S4). The putative targets were uploaded to the Metascape platform and the PPI relationship was determined. The network diagram contains 274 nodes and 3696 edges. The topology of the network diagram was analyzed under the following filter conditions: BC ≥ 0.016, CC ≥ 0.563, and degree ≥80 (fourfold the median). A core target network with 13 nodes and 71 edges was obtained (Fig. 1D, Table 2).

**Table 2:** The PPI network topological characteristics of top 13 major targets.

Gene names	Protein names	Betweenness Centrality	Closeness Centrality	Degree
MAPK3	MAP kinase-activated protein kinase 3	0.032	0.616	109
MAPK1	Mitogen-activated protein kinase 1	0.027	0.609	108
EGFR	Epidermal growth factor receptor	0.033	0.604	101
MYC	Myc proto-oncogene protein	0.049	0.601	101
JUN	Transcription factor AP-1	0.038	0.587	96
TP53	Cellular tumor antigen p53	0.027	0.582	95
HSP90AA1	Heat shock protein HSP 90-alpha	0.027	0.587	93
RELA	Transcription factor p65	0.024	0.585	91
SRC	Proto-oncogene tyrosine-protein kinase Src	0.027	0.578	89
MAPK14	Mitogen-activated protein kinase 14	0.016	0.574	85
STAT3	Signal transducer and activator of transcription 3	0.039	0.463	84
AKT1	RAC-alpha serine/threonine-protein kinase	0.049	0.482	83
PRKCA	Protein kinase C alpha type	0.026	0.563	82

### 3.3 GO and pathway enrichment analysis of QDDHG on DKD

**Fig. 2:**
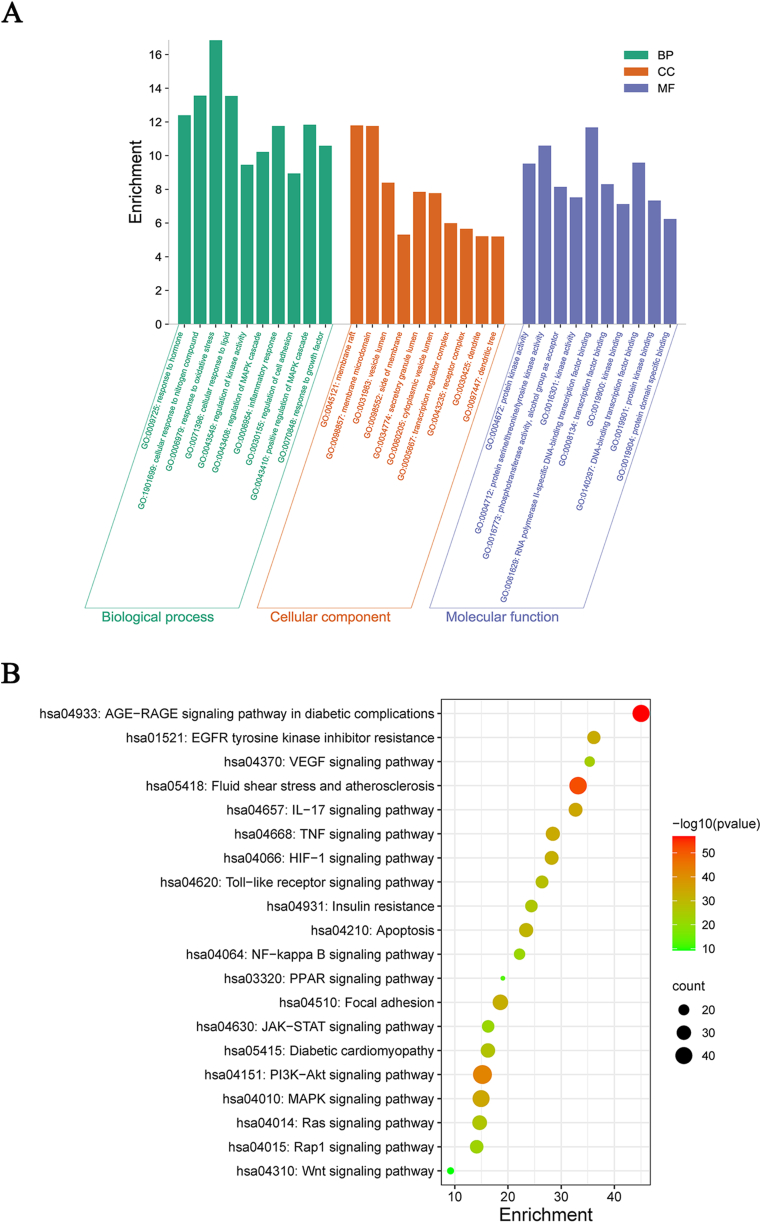
GO and KEGG enrichment of 275 common genes. **(A)** The top 10 GO terms including BP (biological process), CC (cellular component), and MF (molecular function) with adjusted p-values <0.01 were presented in a barplot. (**B)** KEGG enrichment of common genes between QDDHG and DKD.

The GO analysis returned a total of three thousand and two items, including two thousand five hundred and eighty-four items for biological processes (BP), primarily relating to response to hormone, response to oxidative stress, cellular response to lipid, regulation of kinase activity, regulation of MAPK cascade, response to growth factor. One hundred and fifty-five items for cellular component (CC) were identified, primarily relating to membrane raft, dendrite, membrane microdomain, vesicle lumen, side of membrane. Two hundred and sixty-three items were identified for molecular function (MF), primarily related to protein kinase activity, protein serine or threonine or tyrosine kinase activity, phosphotransferase activity (Fig. 2A, Supplementary Table S5).

KEGG pathway enrichment of two hundred and twenty-two pathways were found, primarily relating to the AGE-RAGE in diabetic complications, PI3K-Akt, MAPK, TNF, EGFR tyrosine kinase inhibitor resistance, HIF-1, VEGF, Rap1, NF-kappa B, and JAK-STAT (Fig. 2B, Supplementary Table S6).

### 3.4 Eighteen core active compounds of QDDHG on DKD

**Fig. 3:**
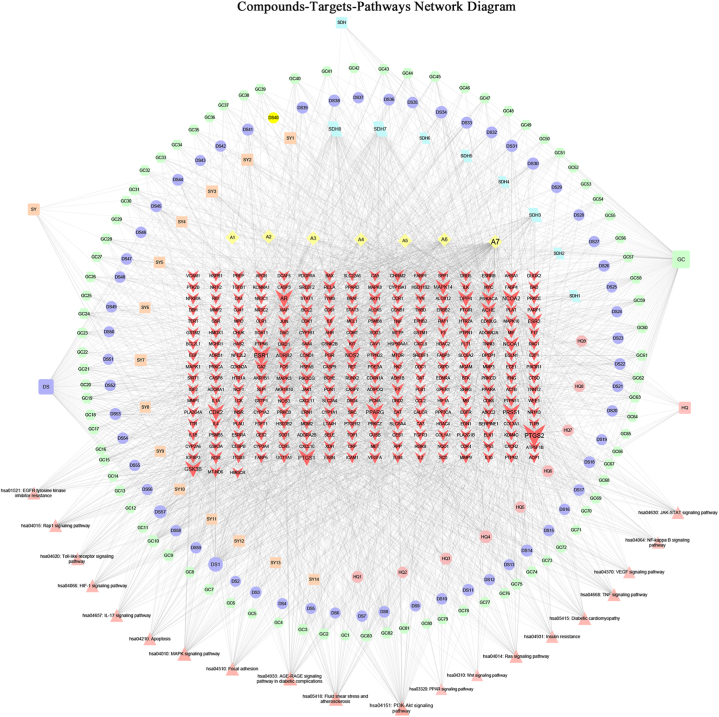
Compounds-targets-pathways network including 480 nodes and 2722 edges. HQ, DS, SHD, SY, and GC refer to Huangqi, Danshen, Dihuang, Shanyao, and Gancao. The different colors and shapes represent drugs, active compounds, genes, and pathways, respectively. In the network, the importance of the genes was expressed by the font size. The yellow diamonds A1-A7 are the common components of the drug that is mairin, jaranol, isorhamnetin, formononetin, calycosin, kaempferol, quercetin in order. The red “V" is the gene corresponding to the active ingredient of the drug. The red triangle is the pathway. (For interpretation of the references to color in this figure legend, the reader is referred to the Web version of this article.)

The compounds-targets-pathways network contained 480 nodes and 2772 edges. In figure, HQ, DS, SDH, SY, and GC refer to Huangqi, Danshen, Dihuang, Shanyao, and Gancao, respectively. The connection between the nodes indicates the targeting relationship between the active compounds, target, and pathway. The greater the number of connections, the larger the font of the node, the more important it is in the network (Fig. 3, Supplementary Table S7).

The network was analyzed by Network Analyzer with the following filter conditions: BC ≥ 0.00068, CC ≥ 0.3731, and degree greater than or equal to 20. In total, eighteen key chemical compounds in the network were screened (Table 3). These high-degree active compounds may play important roles in the treatment of DKD.

**Table 3:** Topological characteristics of 18 active compounds.

Chemical name	Molecular Formula	Betweenness Centrality	Closeness Centrality	Degree	Source of herbal medicines
Quercetin	C15H10O7	0.203	0.468	232	Huangqi, Gancao
Kaempferol	C15H10O6	0.044	0.418	102	Huangqi, Gancao
7-isoquinolinol	C9H7NO	0.097	0.378	56	Dihuang
Ferulic acid methyl ester	C11H12O4	0.088	0.41	55	Dihuang
Isorhamnetin	C16H12O7	0.011	0.4	54	Huangqi, Gancao
Formononetin	C16H12O4	0.017	0.396	53	Huangqi, Gancao
Luteolin	C15H10O6	0.042	0.414	50	Danshen
Rehmapicrogenin	C10H16O3	0.085	0.389	44	Dihuang
Calycosin	C16H12O5	0.002	0.392	34	Huangqi, Gancao
Naringenin	C15H12O5	0.043	0.395	31	Gancao
Tanshinone iia	C19H18O3	0.024	0.383	29	Danshen
3′,7-Dihydroxy-4′,6-Dimethoxyisoflavone	C17H14O6	0.024	0.386	24	Gancao
7-Methoxy-2-methyl isoflavone	C17H14O3	0.007	0.397	24	Gancao
Pinocembrin	C15H12O4	0.016	0.385	23	Gancao
7-*O*-methylisomucronulatol	C18H20O5	0.005	0.395	23	Huangqi
Licochalcone A	C21H22O4	0.006	0.395	21	Gancao
Jaranol	C17H14O6	0.001	0.377	20	Huangqi, Gancao
Dihydrotanshinlactone	C18H14O3	0.003	0.388	20	Danshen

### 3.5 Validation of binding

**Fig. 4:**
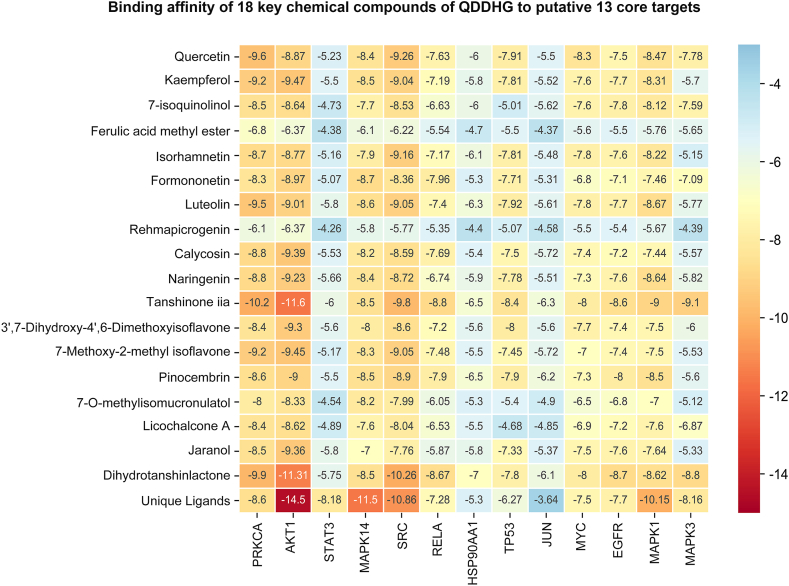
The molecular docking of 18 active compounds and 13 target proteins. The lateral axis is the target protein and the vertical axis is the active ingredient. A more intense blue color represents greater binding energy, and a more intense red color represents a smaller binding affinity. The lower binding energy means better binding effect between the active component and protein. (For interpretation of the references to color in this figure legend, the reader is referred to the Web version of this article.)

Molecular docking was performed between 18 active compounds and 13 proteins. The protein, PDB-ID, and Unique Ligands data are shown in Table 4. A matrix heat map was drawn with the binding energy value, as shown in Fig. 4. When the binding energy is less than or equal to −5.0 kJ mol^−1^, it indicates that the binding of the active component and protein is feasible. The results indicated that 235 docking effects were feasible.

**Table 4:** Protein, PDB-ID, and unique ligands.

Protein	PDB-ID	Unique Ligands
PRKCA	3IW4	LW4
AKT1	3O96	IQO
STAT3	6NJS	KQV
MAPK14	7BDO	TBK
SRC	6E6E	HVY
RELA	6nv2	0V4
HSP90AA1	7RY1	MDC
TP53	5O1h	9 GN
JUN	5FV8	JEF
MYC	5W77	9WP
EGFR	3IKA	0UN
MAPK1	1TVO	FRZ
MAPK3	6GES	6H3

### 3.6 Identification of the chemical components in QDDHG

**Fig. 5:**
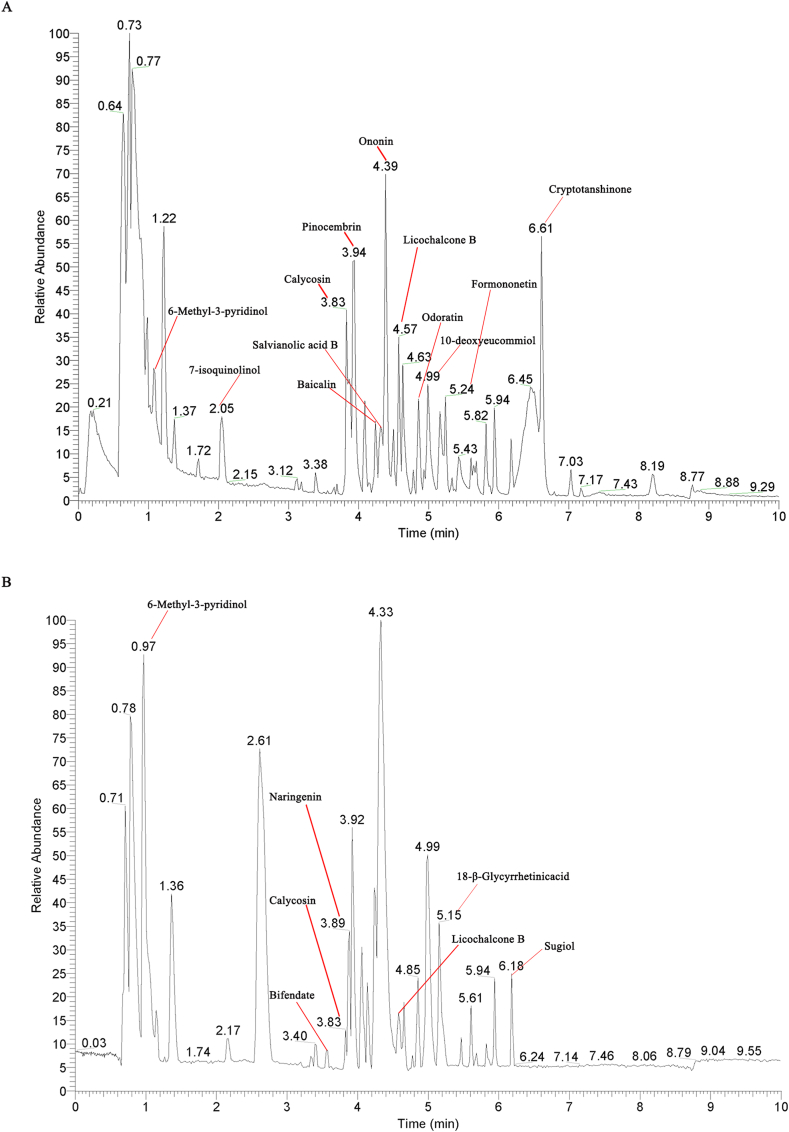
The main components of QDDHG were examined by UHPLC–MS/MS. **(A)** The ion chromatography in positive ion modes. **(B)** The ion chromatography in negative ion modes.

According to the peak integration, retention time, peak alignment, peak extraction and peak area, there have 16 components of QDDHG was detected, namely, 6-Methyl-3-pyridinol, 7-isoquinolinol, calycosin, naringenin, pinocembrin, baicalin, formononetin, licochalcone B, 3′,7-dihydroxy-4′,6-dimethoxyisoflavone (odoratin),10-deoxyeucommiol, cryptotanshinone, salvianolic acid B, ononin, bifendate, sugiol and 18-β-glycyrrhetinicacid (Fig. 5).

### 3.7 QDDHG decrease the FBG, Scr, BUN and 24 h urine albumin

**Fig. 6:**
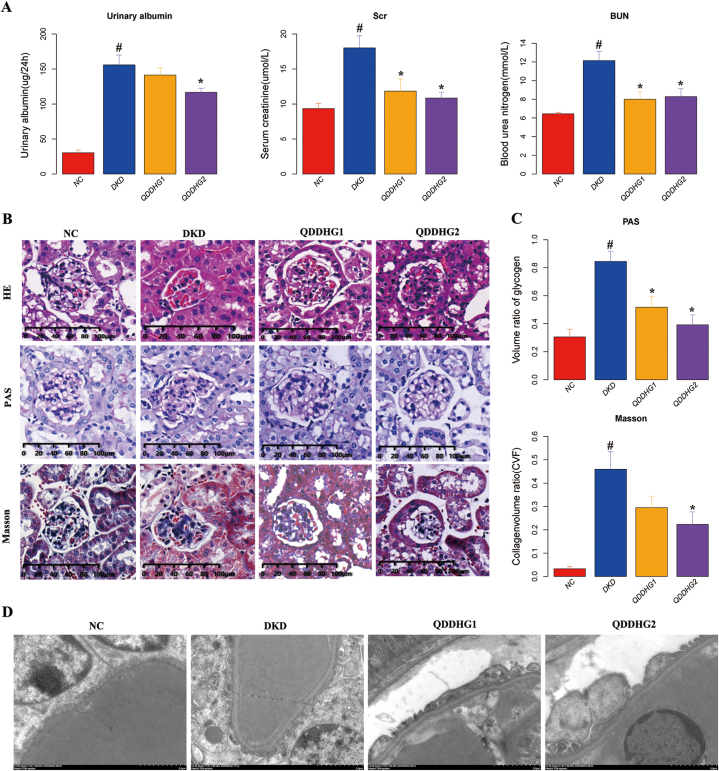
The effect of QDDHG to treat the db/db mice. **(A)** The Effects of QDDHG on 24 h urinary albumin (UALB), serum creatinine (Scr), blood urea nitrogen (BUN). Values are demonstrated as Means ± SEM (n = 6 per group). ^#^p < 0.05 vs NC group; *p < 0.05 vs DKD group. (B) Representative morphological micrographs of kidney tissue stained with HE, PAS, Masson after 16 weeks. **(C)** Bar chart showing the volume ratio of glomerular glycogen with PAS and glomerular collagen volume with Masson staining. Values are demonstrated as Means ± SEM (n = 3 per group). ^#^p < 0.05 vs NC group; *p < 0.05 vs DKD group. **(D)** Representative ultrastructural changes in renal tissue observed with TEM.

**Figure S1:**
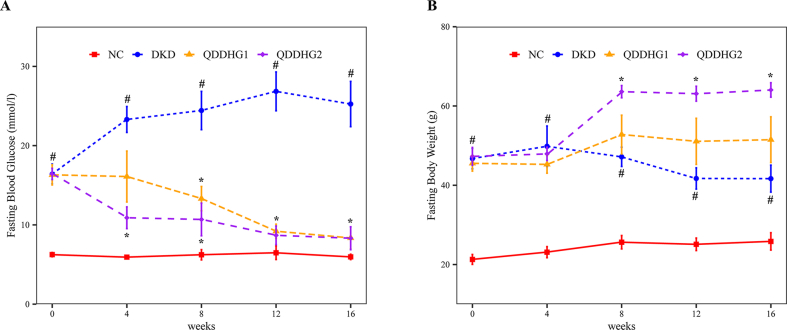
The effects of QDDHG on fasting blood glucose (A) and body weight (B) in each group of mice. Values are demonstrated as Means ± SEM (n = 6 per group). #p < 0.05 vs NC group; *p < 0.05 vs DKD group.

During the experiment, fasting blood glucose declined in the QDDHG1 and QDDHG2 groups (Fig. S1 A). The QDDHG2 group mice displayed a gradual increase in body weight compared with the DKD group mice (Fig. S1 B). After 16 weeks of treatment, QDDHG1 group induced a marked decrease in the serum creatinine (Scr), blood urea nitrogen (BUN) respectively, compared with those in DKD group. In contrast, QDDHG1 group no statistical differences were observed in the 24 h urine albumin (Alb), compared with DKD group mice. However, in QDDHG2 group mice, significant differences in the 24 h urine albumin, Scr, BUN levels were observed, relative to the DKD group mice (Fig. 6A).

### 3.8 QDDHG protected renal structure

Glomerular lesions, including mesangial matrix proliferation, decreased numbers of endothelial cells and mesangial cells, and nodular sclerosis were observed in HE-stained tissue from db/db mice. QDDHG1 and QDDHG2 alleviated pathological alterations of the glomerulus in db/db mice, as revealed by histological examination (Fig. 6B). In glomeruli, Glycogen deposition was measured with PAS staining, and quantitative analysis performed to reveal the severity of glomerulosclerosis, which was significantly improved upon QDDHG1 and QDDHG2 treatments group, relative to DKD group mice. Moreover, quantitative analysis disclosed significantly lower intensity of glycogen deposition in QDDHG1 and QDDHG2 group mice than that in DKD group mice. Since extracellular matrix accumulation is an important indicator of glomerulosclerosis, collagen was showed using Masson staining. Quantitative analysis revealed collagen accumulation within the glomerular area, which was reduced in QDDHG2 (Fig. 6C).

TEM revealed foot processes with a tight arrangement in NC group, these were tightly apposed with each other, and filtration slits were narrow. In contrast, slits between foot processes were wide and foot processes with clearance leakage were observed in DKD group, compared to QDDHG1 and QDDHG2 group. The thickness of the GBM in db/db mice was displayed an increase compared with QDDHG1 and QDDHG2 group (Fig. 6D).

### 3.9 QDDHG restored nephrin expression of glomerular podocytes in renal tissue

**Fig. 7:**
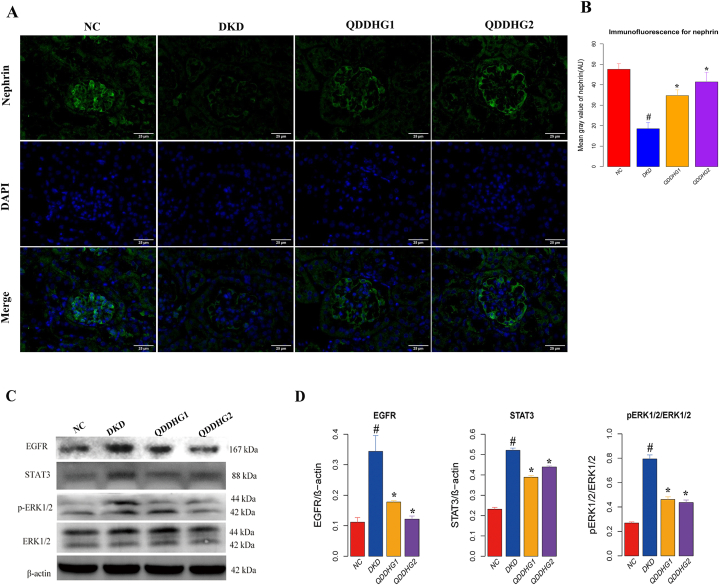
The influence of QDDHG on the expression of nephrin by immunofluorescence and protein expressionof EGFR, STAT3, ERK1/2 and pERK1/2 in renal tissues by Western blot analysis. **(A)** Immunofluorescence staining of nephrin in the four groups. **(B)** The mean gray value of nephrin by immunofluorescence. **(C)** Expressions of EGFR, STAT3, ERK1/2 and pERK1/2 protein in the four groups**,** as detected by Westernblot analysis. **(D)** The relative levels of protein expression EGFR, STAT3, ERK and pERK in renal tissues with β-actin expression used as the inner control. Values are presented as Means ± SEM. ^#^p < 0.05 vs NC group; *p < 0.05 vs DKD group.

In addition to the pathological lesions, fewer glomerular podocytes were also observed in the DKD group; these are known to contribute to the development of DKD. Nephrin, a podocyte-specific marker, was used to verify whether podocyte loss occurred in DKD mice (Fig. 7A). As shown in Fig. 7A, the nephrin protein of podocytes was downregulated in the DKD group compared with the NC group but was upregulated in the QDDHG1 and QDDHG2 groups compared with the DKD group.

### 3.10 QDDHG Suppressed the expression of EGFR, STAT3 and pERK1/2 in renal tissue

In order to further verify the mechanism predicted by network pharmacology, the protein expression of EGFR, STAT3, ERK and pERK1/2 were adopted. The Western blot analysis revealed significant upregulation in the expression of EGFR, STAT3 and pERK1/2 proteins in DKD group, compared with NC group, using β-actin expression as the inner control. Treatment with QDDHG1 and QDDHG2 induced a decrease in protein expression of EGFR, STAT3 and pERK1/2 in db/db mice, compared with DKD group (Fig. 7C and D).

## 4 Discussion

In the previous study, QDDHG was proved to be a promising drug in the aspect of reducing albuminuria and protecting the renal function [10]. However, the exact mechanism of QDDHG on DKD remains to be clarified. Network pharmacology offers a new perspective on drug discovery [16] and has proven to be a comprehensive means to effectively uncover the complicated network relationships between the active ingredients of TCM formulas and their potential mechanisms [17].

### 4.1 The roles of potential targets on DKD

Potential targets of QDDHG on DKD were identified by network pharmacology. Studies show that intervention of EGFR tyrosine kinase inhibitors in model animals can postpone the progression of DKD [18]. When the EGFR of podocyte was deleted specifically, glomerular injury caused by streptozotocin was ameliorated [19]. MAPK3 and MAPK1, also known as ERK1 and ERK2 respectively. Studies indicate that the activation of ERK1/2 pathway in the human glomerulopathies was related with renal fibrosis and dysfunction [20], while other study found that the downregulation of phosphorylated ERK1/2 expression level and upregulation of phosphorylated JNK expression levels were associated with apoptosis in rat kidney with diabetes [21]. STAT3 is a signal transducer and plays critical roles in cell growth, differentiation, and inflammatory response [22]. Activated STAT3 was a key factor in the development of renal fibrosis caused by hyperuricemia. The inhibition of STAT3 ameliorated kidney function and alleviated renal fibrosis in the hyperuricemic nephropathy mice [23]. AKT belongs to a serine/threonine kinase that is a crucial regulator of cell apoptosis, proliferation, migration, metabolism, and angiogenesis [24]. In the kidneys, AKT is involved in the proliferation and activation of tubular epithelial cells, glomerular mesangial cells, and interstitial fibroblasts during the development of renal fibrosis [24].

### 4.2 The action of putative key components on DKD

In this study, 18 key ingredients were predicted as the main components in QDDHG for DKD, including quercetin, kaempferol, 7-isoquinolinol, ferulic acid methyl ester, formononetin, isorhamnetin, luteolin, rehmapicrogenin, calycosin, naringenin, tanshinone IIA, 3′,7-dihydroxy-4’,6-dimethoxy isoflavone, pinocembrin, 7-methoxy-2-methyl isoflavone, 7-*O*-methylisomucronulatol, licochalcone A, dihydrotanshinlactone and jaranol.

Quercetin can decrease the levels of albumin in rat urine, increase the excretion rate of urinary creatinine and urea nitrogen, and improves renal function [25]. Kaempferol can inhibit the expression of DKD markers such as CTGF, TGF-β1, and fibronectin in DKD kidney tissues, thereby improving the renal fibrosis [26]. Experiments on the db/db mice have demonstrated that formononetin can improve renal oxidative stress and prevent the progression of renal fibrosis by activating the Nrf2-ARE signal pathway and increasing SIRT1 levels [27]. Isorhamnetin exerts protective effects on the kidneys of streptozotocin- and high-fat diet-induced DKD animals by regulating of autophagy-related genes [28]. Luteolin exerts anti-inflammatory and anti-oxidative stress effects. Tanshinone IIA treatment can improve glutathione-mediated detoxification pathway to oppose the inflammation and excess oxidative stress triggered by high glucose [29]. Luteolin can improve glomerulosclerosis and renal interstitial fibrosis in the db/db mice mainly by inhibiting the STAT3 pathway [30]. In vitro, rehmapicrogenin exhibits nitric oxide inhibitory activities and anti-inflammatory action by inhibiting iNOS, IL-6 and COX-2 [31]. *In vivo* and *in vitro* studies have revealed that calycosin inhibits diabetes-induced kidney inflammation, mainly by inhibiting NF-κB p65 phosphorylation [32]. Naringenin treatment can reduce plasma glucose levels and blood urea nitrogen, increase creatinine clearance [33]. Licochalcone A can significantly lower the insulin resistance index, and lipid levels in mice with diabetes mice [34]. In addition, the effects of ferulic acid methyl ester, rehmapicrogenin, 7-methoxy-2-methyl isoflavone, 7-*O*-methylisomucronulatol, 3′,7-dihydroxy-4′,6-dimethoxyisoflavone, pinocembrin, dihydrotanshinlactone and jaranol in DKD have not been reported in the literature. Therefore, future research is needed on these compounds and their role in treating DKD.

Furthermore, the molecular docking was employed to verify active compounds and potential proteins of QDDHG to treat the DKD. The binding energy with a more negative represents a more stable complexes of the bound ligand-protein conformation [35]. The binding affinity shows that tanshinone IIA with PPKCA, AKT1, STAT3, RELA, TP53, HSP90AA1, JUN, MAPK1 and MAPK3, formononetin with MAPK14, dihydrotanshinlactone with SRC and EGFR, pinocembrin, with HSP90AA1, Quercetin with MYC were exhibiting a significant minimum binding affinity among active compounds and target proteins. It was speculated that the above compounds might exert a more crucial role in the treatment of QDDHG on DKD.

### 4.3 GO and KEGG pathway enrichment of QDDHG on DKD

Go enrichment shows that the top 5 most significant terms of biological process included response to hormone (GO:0009725), cellular response to nitrogen compound (GO:1901699), response to oxidative stress (GO:0006979), cellular response to lipid (GO:0071396), regulation of kinase activity (GO:0043549). In the biological process, STAT3 was involved of response to hormone and regulation of kinase activity, EGFR, STAT3, MAPK1 and MAPK3 protein were involved in the process of cellular response to nitrogen compound, EGFR, MAPK1 and MAPK3 protein were participated in the biological process of oxidative stress and cellular response to lipid. From KEGG enrichment analysis, the AGE-RAGE, PI3K-Akt, IL-17, MAPK, TNF, EGFR, HIF-1, Apoptosis, Toll-like receptor, Insulin resistance, VEGF, Rap1, NF-kappa B, JAK-STAT, PPAR and Wnt pathway were displayed as the critical signaling participated in the role of QDDHG on DKD. Especially, STAT3, EGFR, MAPK1 and MAPK3 were extensively involved in the above multiple biological processes and pathways.

### 4.4 Components and animal experimental verification of QDDHG on DKD

Identification of the chemical components of QDDHG was performed by UHPLC-MS/MS. The results showed that 13 ingredients predicted by network pharmacology were also confirmed by UHPLC-MS/MS. Moreover, six of the key ingredients predicted by network pharmacology were also verified by UHPLC-MS/MS, namely, calycosin, 7-isoquinolinol, naringenin, pinocembrin, formononetin, 3′,7-Dihydroxy-4′,6-dimethoxyisoflavone (odoratin). In view of the role of these components, we evaluate the overall effect of QDDHG on DKD treatment via animal experiments.

Traditionally, urinary albumin, Scr and BUN levels have been used to monitor the progression of DKD [36]. In this study, the alleviated urinary albumin, Scr and BUN levels reflect the effects of the QDDHG treatment in preventing renal lesions. It is widely known that pathological glomerular changes are one of the representative features of DKD [37]. The glomerular basement membrane, mesangial matrix proliferation, number of endothelial and mesangial cells, and nodular sclerosis were markedly improved by the QDDHG treatment in this study. Moreover, PAS and Masson's trichrome staining, indicating the severity of glomerulosclerosis, was significantly alleviated in the DKD mice subjected to the QDDHG treatment. Consistent with these observations, the TEM data verified the tight arrangement of foot processes and filtration slits in the db/db mice that underwent QDDHG treatment. Podocyte injury plays an important role in the progression of DKD. The expression of nephrin (a biomarker of podocytes) is a marker of normal podocytes and decreased nephrin expression is closely correlated with DKD progression [38]. QDDHG ameliorated podocyte injury and decreased proteinuria by maintaining podocyte nephrin expression in db/db mice.

Subsequently, we utilized animal experiments to verify the mechanism of QDDHG predicted by the GO and KEGG enrichment. The protein expressions of EGFR, STAT3, ERK1/2 in the QDDHG1-and QDDHG2-treated db/db mice demonstrated an obvious reduction, compared with that in the untreated db/db mice. Study from the literature also shown that regulating the STAT3 pathway improves DKD [39]. The deletion of selective podocyte EGFR led to marked reduction in albuminuria and glomerulosclerosis, and relative podocyte preservation in db/db mice [40], and regulating the activity of EGFR reduced the production of ROS [41]. Also, the proliferation and oxidative stress were inhibited in high glucose-induced human glomerular mesangial cells by the inactivation of ERK signaling pathway [42]. Furthermore, the increased extracellular ERK1/2 activity in podocytes, which contributed to an increase in cholesterol influx and autophagy inhibition [43]. STAT3, EGFR, ERK1 and ERK2 were identified as key targets of QDDHG on DKD. The animal experimental results are consistent with network pharmacology and literature. EGFR, ERK1 and ERK2 were involved in the biological process of oxidative stress and lipid metabolism, and play the crucial role of QDDHG on the DKD treatment [40,42]. This is the first time to unveil the action and mechanisms of QDDHG on the DKD treatment.

## 5 Limitations

However, there are still some limitations in this study. we did perform UHPLC-MS/MS analysis on the active compounds of the QDDHG, but did not verify the single drug or components *in vivo* and *in vitro* experiments. Because of TCM action based on synergy and interaction of multiple components, experimental research performed on a single drug or components not necessarily increase the evidence of QDDHG on DKD. In addition, there are other potential active compounds and putative pathway from the KEGG enrichment, more *in vivo* experiments should be conducted to elucidate the action and mechanisms.

## Conclusion

The mechanism of QDDHG on reducing the levels of albuminuria and protecting the renal function mainly through inhibited the expression of EGFR, STAT3 and pERK in DKD.

## Author contributions

LX, XSC and XSZ designed the study. YLL, YX and PPJ performed the data analysis. LX and XSC drafted the manuscript. XFJ, HLY and DS revised the manuscript. All authors gave approval for this study to be published.

## Funding

This research was supported by the 10.13039/501100001809National Natural Science Foundation of China (81804030), the Fellowship of 10.13039/501100002858China Postdoctoral Science Foundation (2022M711347, 2022M711536), the 10.13039/501100003453Natural Science Foundation of Guangdong Province, China (2018030310451, 2018A0303130320), the 10.13039/501100010883Traditional Chinese Medicine Bureau of Guangdong Province, China (20222203) and the Major project of University of Chinese Academy of Science-Shenzhen Hospital , China (HRF-2021003).

## Data availability statement

The data used to support the findings of this study are included within the manuscript and supplementary information files.

## Declaration of competing interest

The authors declare that they have no known competing financial interests or personal relationships that could have appeared to influence the work reported in this paper.

## Footnotes

Appendix A Supplementary data to this article can be found online at https://doi.org/10.1016/j.heliyon.2023.e21714.
